# Supplementary material for: Apelin signaling drives vascular endothelial cells toward a pro-angiogenic state
Source: eLife. 2020 Sep 21;9:e55589. doi: 10.7554/eLife.55589 (PMC7567607; doi:10.7554/eLife.55589)
Supplement: Figure 4—source data 1. [file elife-55589-fig4-data1.docx]

**Figure 4—source data 1.** Ct values of RT-qPCR

| cells | gene | sample | dCt value (experiment) | dCt value (control) |
| --- | --- | --- | --- | --- |
| HUVEC | *APLN* | DAPT | 3.61 | 4.45 |
| HUVEC | *APLN* | DAPT | 3.94 | 4.87 |
| HUVEC | *APLN* | Dll4 6 h | 4.10 | 0.89 |
| HUVEC | *APLN* | Dll4 6 h | 5.44 | 2.74 |
| HUVEC | *APLN* | Dll4 12 h | 3.08 | 1.57 |
| HUVEC | *APLN* | Dll4 12 h | 2.50 | 1.45 |
| HUVEC | *APLN* | Dll4 24 h | 4.78 | 3.82 |
| HUVEC | *APLN* | Dll4 24 h | 4.48 | 3.48 |
| HUVEC | *APLN* | Dll4 24 h | 6.11 | 5.60 |
| HUVEC | *APLN* | Dll4 24 h | 5.92 | 5.42 |
| HUVEC | *PFKFB3* | *APLN* + *APLNR* siRNA | 4.62 | 4.00 |
| HUVEC | *PFKFB3* | *APLN* + *APLNR* siRNA | 4.51 | 3.92 |
| HUVEC | *PFKFB3* | *APLN* + *APLNR* siRNA | 4.74 | 4.09 |
